# Spontaneous Liquefaction of Solid Metal–Liquid Metal Interfaces in Colloidal Binary Alloys

**DOI:** 10.1002/advs.202400147

**Published:** 2024-05-05

**Authors:** Caiden J. Parker, Karma Zuraiqi, Vaishnavi Krishnamurthi, Edwin LH Mayes, Pierre H. A. Vaillant, Syeda Saba Fatima, Karolina Matuszek, Jianbo Tang, Kourosh Kalantar‐Zadeh, Nastaran Meftahi, Chris F. McConville, Aaron Elbourne, Salvy P. Russo, Andrew J. Christofferson, Ken Chiang, Torben Daeneke

**Affiliations:** ^1^ School of Engineering RMIT University Melbourne 3001 Australia; ^2^ School of Science RMIT University Melbourne 3001 Australia; ^3^ School of Chemistry Monash University Clayton 3800 Australia; ^4^ School of Engineering University of New South Wales (UNSW) Sydney 2052 Australia; ^5^ School of Chemical and Biomolecular Engineering The University of Sydney Sydney 2008 Australia; ^6^ ARC Centre of Excellence in Exciton Science School of Science RMIT University Melbourne 3001 Australia; ^7^ Department of Physics University of Warwick Coventry CV4 7AL UK

**Keywords:** binary alloys, CuGa_2_, gallium colloid, liquefaction, liquid metal, nanodroplets

## Abstract

Crystallization of alloys from a molten state is a fundamental process underpinning metallurgy. Here the direct imaging of an intermetallic precipitation reaction at equilibrium in a liquid‐metal environment is demonstrated. It is shown that the outer layers of a solidified intermetallic are surprisingly unstable to the depths of several nanometers, fluctuating between a crystalline and a liquid state. This effect, referred to herein as crystal interface liquefaction, is observed at remarkably low temperatures and results in highly unstable crystal interfaces at temperatures exceeding 200 K below the bulk melting point of the solid. In general, any liquefaction process would occur at or close to the formal melting point of a solid, thus differentiating the observed liquefaction phenomenon from other processes such as surface pre‐melting or conventional bulk melting. Crystal interface liquefaction is observed in a variety of binary alloy systems and as such, the findings may impact the understanding of crystallization and solidification processes in metallic systems and alloys more generally.

## Graphical abstract

The phenomenon of solid metal surface liquefaction within liquid metal environments, represents an intriguing equilibrium state, defying common expectations by oscillating between a solid and liquid phase at temperatures significantly below the intermetallic compounds' conventional melting point. This discovery promises to advance the understanding of fundamental chemistry in metallic systems, offering insights into their behavior and potential applications.

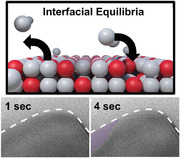

## 1 Introduction

Metals and alloys are one of the largest and most integral classes of materials that underpin society, finding applications in almost everything one encounters in daily life.^[^ ^1^ ^]^ Most alloys begin their journey as a molten metal, which then cools to form a solid. The process of solidification is crucial as it dictates the final physical, chemical and mechanical properties, which are profoundly impacted by the final crystalline structure, size and shape.^[^ ^1^, ^2^ ^]^

In contrast to crystallization processes that occur in covalent or ionic solvents, surprisingly little is known about the solvation and colloidal chemistry within liquid metal environments.^[^ ^3^, ^4^, ^5^, ^6^, ^7^, ^8^ ^]^ This is largely due to the opaque nature of molten metals to most light‐based characterization like UV–vis, Raman, and infrared spectroscopy.^[^ ^1^, ^9^, ^10^, ^11^, ^12^, ^13^, ^14^, ^15^ ^]^ Techniques based on hard X‐rays including X‐ray reflectometry, X‐ray Absorption Spectroscopy, and X‐ray powder diffraction can provide insights, however, these techniques are limited due to their comparatively long data collection times and high photon energy, meaning they predominantly provide long term averaged structural and oxidation state information averaged across a bulk sample.^[^ ^15^, ^16^, ^17^, ^18^, ^19^, ^20^ ^]^ As such, time and spatially resolved measurements have been difficult to conduct. As a result, the important early stages of solidification, where the metal begins to crystallize, while a large fraction of the material remains liquid, is still relatively poorly understood.^[^ ^21^, ^22^, ^23^ ^]^ This work focuses on exploring solid intermetallic particles surrounded by the liquid metal mother liquor from which they precipitated. This was achieved using a transmission electron microscope (TEM) equipped with in situ heating and selecting alloy systems that melt at accessible, low temperatures. In this work, we report on the observation of unexpectedly dynamic interfaces between the solid and liquid metal fractions. It is shown that the outer layers of the crystal spontaneously become liquid and then recrystallize at room temperature. TEM‐based video recording confirms that this behavior is persistent, remaining observable several days after sample preparation and at temperatures far below the formal melting point of the solid. This distinguishes the observed phenomenon from established processes such as surface pre‐melting.^[^ ^13^, ^24^, ^25^ ^]^ Furthermore, this effect can extend deep into the solid lattice, causing up to 10 nm of the solid (≈50 atom layers) to spontaneously transition temporarily into a liquid state. As such, this interface instability of a colloidal solid metal in a liquid metal solvent environment is found to extend much further into the crystal, particularly when compared with what has recently been observed for solvation, crystallization, and Ostwald ripening processes in conventional solvents. For example, TEM‐based video observations of crystalline palladium hydrate formation and the crystallization and ripening of cadmium sulfide nanocrystals, show that only the outer 2–5 atom layers are mobile for those systems.^[^ ^12^, ^26^ ^]^ In this paper, we show that the surprising depth of the observed crystal interface liquefaction is likely linked to the incorporation of the metallic solvent metal into the crystalline lattice of the precipitated intermetallic compound.

## 2 Results and Discussion

### 2.1 Surface Liquefaction of the Cu‐Ga System

**Figure 1:**
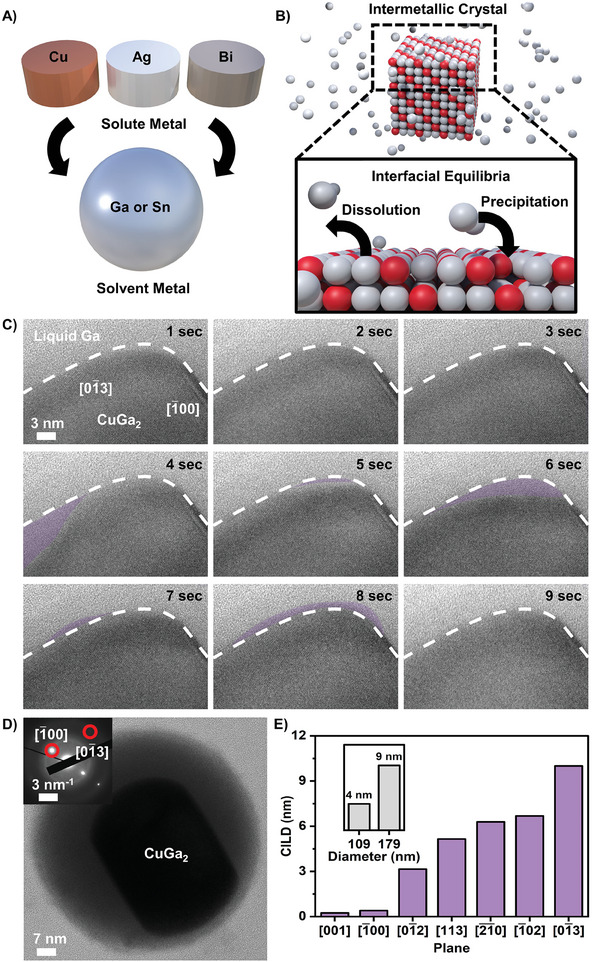
A) Material selection process showing the solute metals (top) and the liquid metal solvents (bottom), B) Schematic of an intermetallic colloidal particle undergoing an equilibrium reaction whereby liquid metal atoms migrate into solution (dissolution) to then later be replaced by another liquid metal atom from the bulk solution (precipitation). C) Time series for dynamic movement of the CuGa_2_ crystal interface (i.e., crystal interface liquefaction) in a liquid metal environment conducted at 25 °C. The outlined areas highlight where the crystal boundary moved (shaded purple). D) Bright‐field TEM image showcasing the Cu‐Ga metal in metal colloid structure, with selected area electron diffraction (SAED) shown as an inset, and E) Graphical representation demonstrating facet dependence and size dependence (inset) of the crystal interface liquefaction depths (CILD) all conducted at 25 °C. A version of this figure without purple shading is provided in the supplementary information (Figure S11, Supporting Information).

Binary alloy systems consisting predominantly of a low‐melting‐point post transition metal such as gallium or tin and approximately 5–15 wt.% of another dissolved metal, were synthesized using a thermomechanical alloying method.^[^ ^27^ ^]^ Upon cooling, these alloys create metal‐in‐metal colloids, where solid metal particles are suspended in a liquid metal environment, while the outer surface of the alloy is covered by the formation of a self‐limiting nanometer thick oxide layer determined by the Cabrera‐Mott process.^[^ ^28^, ^29^, ^30^ ^]^ Due to the low melting point of the selected solvent metals, the colloidal stage of the solidifying alloy can be preserved, allowing for detailed observations and investigation **Figure** **1**A,B provides an overview of the systems studied and the types of metals used. To image these systems via TEM, the bulk samples were broken down into 100 to 200 nanometer‐sized droplets by sonication at temperatures above the melting point of the alloys. This ensures the formation of nanodroplets with a homogeneous composition, which on cooling, contain enclosed solid colloidal particles.^[^ ^28^ ^]^ The interface between the liquid metal and the solid colloidal particle was then investigated using high‐resolution TEM imaging and real‐time TEM video recording. Video S1 (Supporting Information), together with additional Videos [Link], [Link], [Link], [Link], [Link], [Link], [Link], [Link] (Supporting Information) are available as part of the electronic supporting information, and we encourage the reader to view these, since they best convey the observed effects. Video S1 (Supporting Information) illustrates a nanodroplet generated from a 5 wt.% copper in a gallium alloy, that upon cooling to room temperature formed a CuGa_2_ intermetallic colloidal particle (See phase diagram Figure S1, Supporting Information).^[^ ^31^ ^]^ It can clearly be seen that the outer atomic layers of the solid intermetallic are perpetually transitioning between a liquid and solid state, engaging in alternating formation and disintegration of a crystalline lattice, without a clear cyclical pattern (Figure 1B). The observed effect is herein referred to as crystal interface liquefaction. Captured images showcasing crystal interface liquefaction for the gallium copper alloy taken over a 9 s interval, can be seen in Figure 1C. The crystalline matter is clearly identifiable due to the presence of a regular lattice. The grey environment surrounding the solid, indicated by the lack of lattice fringes, is not vacuum or the carbon membrane of the TEM grid, but is in fact liquid gallium. An image of the entire nanodroplet captured at a lower magnification is shown in Figure 1D.

The observed effect can be conceptualized as a reversible reaction at equilibrium involving the dissolution and precipitation of an intermetallic. Here we observe a transient crystal boundary at room temperature in the absence of heating (i.e., a static heating/cooling rate of 0 °C/s) with no indication of stable surface formation, even several days after the colloidal particles have been synthesized. As such, the observed crystal interface liquefaction phenomenon cannot simply be explained as a recrystallization or ripening process which would be expected to cease after a stable configuration is reached.

Interestingly, the reported thermal stability limit of the intermetallic (CuGa_2_) is found to be 259 °C.^[^ ^31^, ^32^ ^]^ This was later confirmed via in situ heating experiments within the TEM along with differential scanning calorimetry (DSC) (see Figures S2 and S3 and discussion in Supporting Information along with Video S2, Supporting Information). The DSC measurements show that size‐induced melting temperature depression occurs for the solvent metal (i.e., Ga), while the decomposition of the intermetallic CuGa_2_ occurs at the stability threshold of this phase. This is outlined in the published phase diagram and has been confirmed in recent reports.^[^ ^32^ ^]^ The absence of any significant melting point depression for the CuGa_2_ crystal, despite being in the nanometric regime, is noteworthy and could be associated with the delocalized metallic bond of the surrounding solvent metal counteracting the typically observed size effects. In light of the DSC and in situ heating TEM measurements, the observed surface liquefaction of CuGa_2_ at room temperature does indeed occur at unexpectedly low temperatures, far below the melting point of the solid. It is important to highlight that for the experimental conditions encountered during TEM imaging, beam damage and beam heating are negligible and hence do not play any significant role in these observations (Figure S4, Supporting Information, Discussion).^[^ ^33^ ^]^ The investigated liquid metal droplets can therefore be conceptualized as isothermal closed systems.

A low‐magnification bright field image of a representative metal‐in‐metal colloidal structure is shown in Figure 1D indicating that the solid particle is suspended within the core of the liquid metal droplet from which it precipitated. The boundary of the visible rectangular solid‐core structure is where the dynamic movement occurs, while the overall spherical droplet remains stationary (Video S3, Supporting Information). When assessing the movements of the liquid/solid boundary, it is important to note that the orientation of the solid crystal does not change, as evidenced by the regular crystalline pattern consistently observed across all images. As such, any observed boundary movements do not arise from artefacts linked to the rotation of an irregularly shaped crystal. The selected area electron diffraction (SAED) pattern shown as the inset in Figure 1D, was used to identify the crystal orientation,^[^ ^34^ ^]^ and in turn the crystal facets that exhibit the liquefaction of the crystal interface. By tilting the sample inside the TEM system and aligning it to various crystalline zone axes, it was possible to image different facets inside a nanodroplet. Furthermore, studying droplets with similar sizes that feature colloidal crystals with different orientations, or simply heating the sample and permitting the colloidal particle to recrystallize with random orientation, allowed access to further exposed facets. The magnitude of the boundary shift, or crystal interface liquefaction depths (CILD), was found to be surprisingly large in some cases reaching up to 10 nm for certain crystal facets. This corresponds to >50 atom layers. The [$0\bar{1}3$] facet was found to be the most reactive, whereas the [$\bar{1}00$] exhibited the least dynamic movement. Figure 1E summarizes the facet‐dependent crystal interface liquefaction depths at several interfaces (see Video S4, Supporting Information). Whereas all observed interfaces show liquefaction to varying extents, some orientations are clearly more active than others. This is not surprising since it is well known that certain facets minimize the material's surface energy more effectively than others, which has been exploited to grow both nanoscale and micron‐sized particles with unique morphologies.^[^ ^7^ ^]^

The effect of the overall droplet size was demonstrated in Video S5 (Supporting Information) and is summarized in the inset to Figure 1E. Significant variations in droplet size were also found to impact the crystal interface liquefaction depth. By observing the same [$01\bar{2}$] facet for 179 nm and 109 nm sized droplets this effect was found to be more pronounced for larger particles (Figure 1E inset). This observation can be rationalized by the Laplace pressure, where for small particles, a higher pressure is exerted onto the liquid metal via strong surface tension, resulting in a more stable interface (See Laplace discussion in Supporting Information). As a result of this finding, the study of facet dependency discussed above was carried out on droplets within a narrow size regime between 100–120 nm. While the overall droplet size distribution of the synthesized particles was broad and similar to previous work, care was taken to select similar sized particles for detailed analysis.^[^ ^28^ ^]^

### 2.2 Crystal Interface Liquefaction in Further Metallic Systems

**Figure 2:**
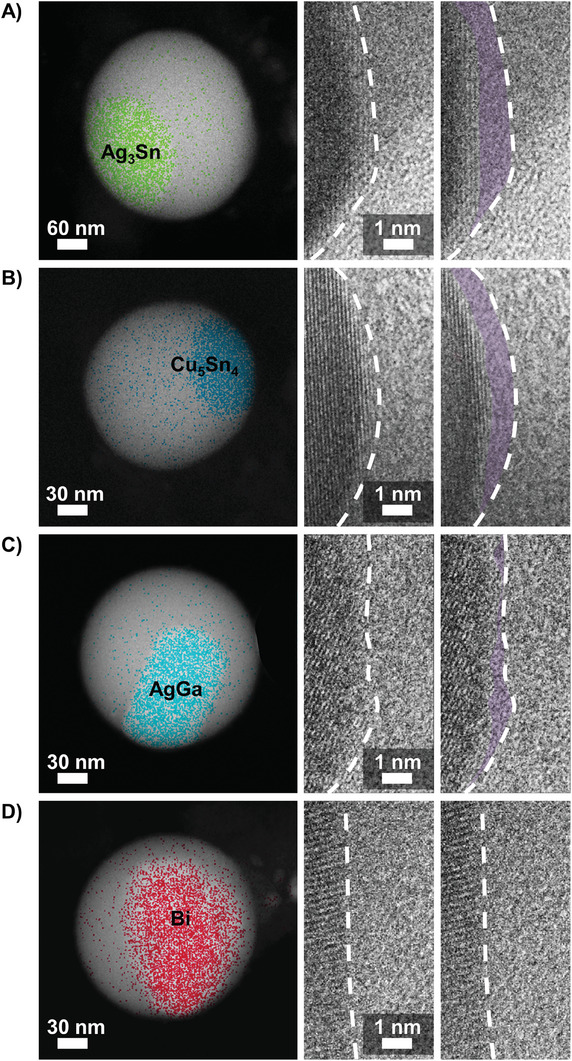
Crystal interface liquefaction investigated in A) Ag‐Sn at 130 °C, B) Cu‐Sn at 130 °C, C) Ag‐Ga at 80 °C, and D) Bi‐Ga at 25 °C. The left column shows dark field images of the studied droplets. The colored elemental map highlights the distribution of the respective added solute metal measured via electron dispersive X‐ray spectroscopy. The composition of the solid colloidal particle has been determined via electron diffraction studies. The middle and right‐hand columns show high‐resolution images of an outer facet exposed to the liquid metal liquor, with the outlined areas in the right‐hand column highlighting areas of change (shaded purple) where the lattice boundary has shifted due to the liquefaction of the crystal interface. A version of this figure without purple shading is provided in the supplementary information (Figure S12, Supporting Information). Care was taken to select nanodroplets with similar size in a 100–120 nm range.

The dynamicity of the solid metal‐liquid metal interface can also be observed for alloys beyond the Cu‐Ga system (Video [Link], [Link], [Link], [Link], Supporting Information). For example, replacing the liquid solvent metal with tin, or the solute metal with silver indicated that it did not stabilize the interface and the dynamic surface layer remains liquid‐like. **Figure** **2**A,B, showcase the tin‐based alloys which were measured after re‐melting the sample inside the TEM using in situ heating, followed by cooling to a temperature of 100–130 °C at which point the tin fraction remained in a liquid state due to supercooling and particle size effects. Once the target temperature was reached for each alloy, the temperature was kept constant.

For the Ag‐Sn system which forms Ag_3_Sn,^[^ ^35^ ^]^ crystal interface liquefaction was observed at elevated temperatures albeit to a lesser extent than for the Cu‐Ga based alloy. The Cu‐Sn system that featured the solid intermetallic Cu_5_Sn_4_^[^ ^36^ ^]^ also exhibited interface liquefaction at temperatures ≈130 °C. It is worth noting that liquefaction can only occur at these high temperatures since the bulk Sn is solid at room temperature. When the Sn solvent is solid, no interfacial liquefaction is observed, as could be expected. The Ag‐Ga system shown in Figure 2C featured crystalline AgGa^[^ ^37^ ^]^ which displayed surface instability only within the outer layers. However, as the system temperature was increased to, and then kept constant at 80 °C, interfacial liquefaction became much more pronounced.

All previously mentioned alloy systems feature the formation of well‐defined intermetallics, where the solvent metal crystallizes together with the solute into a well‐structured lattice. For each of these systems, the intermetallic compounds were identified using Fast Fourier‐Transform (FFT) analysis of high‐resolution TEM images and energy dispersive X‐ray spectroscopy (EDS) as outlined in Figures S5–S7 (Supporting Information) and the associated Supporting Information.

One possible explanation for the observed dynamic motion centers on the incorporated solvent metal within the intermetallic, which could transition from a solid lattice site into the liquid metal phase thus leading to a significant number of vacancies. The formation of vacancies is known to play a key role in both surface and bulk melting, since once the vacancy density exceeds a threshold of ≈10%, the lattice can spontaneously collapse into a liquid state.^[^ ^38^, ^39^, ^40^ ^]^ Following the collapse of the lattice, the supersaturated alloy then recrystallizes spontaneously, leading to the curious situation where a reversible reaction can be observed in real‐time, clearly showing both the forward (precipitation) and reverse (dissolution) reactions within the closed isothermal system of the nanodroplet.

To test whether the incorporation of the solvent metal in the solid colloid is key, a Bi‐Ga alloy was investigated as this is a system that cannot form intermetallic compounds, i.e., in Figure 2D. In this case, the solid core inside the liquid metal droplet consists of elemental bismuth (as described in the supplementary discussion) without the incorporation of gallium in the lattice. Indeed, this system did not show obvious signs of crystal interface liquefaction and instead the observed alloy system was static.

### 2.3 Theoretical Modeling of the Cu‐Ga System

**Figure 3:**
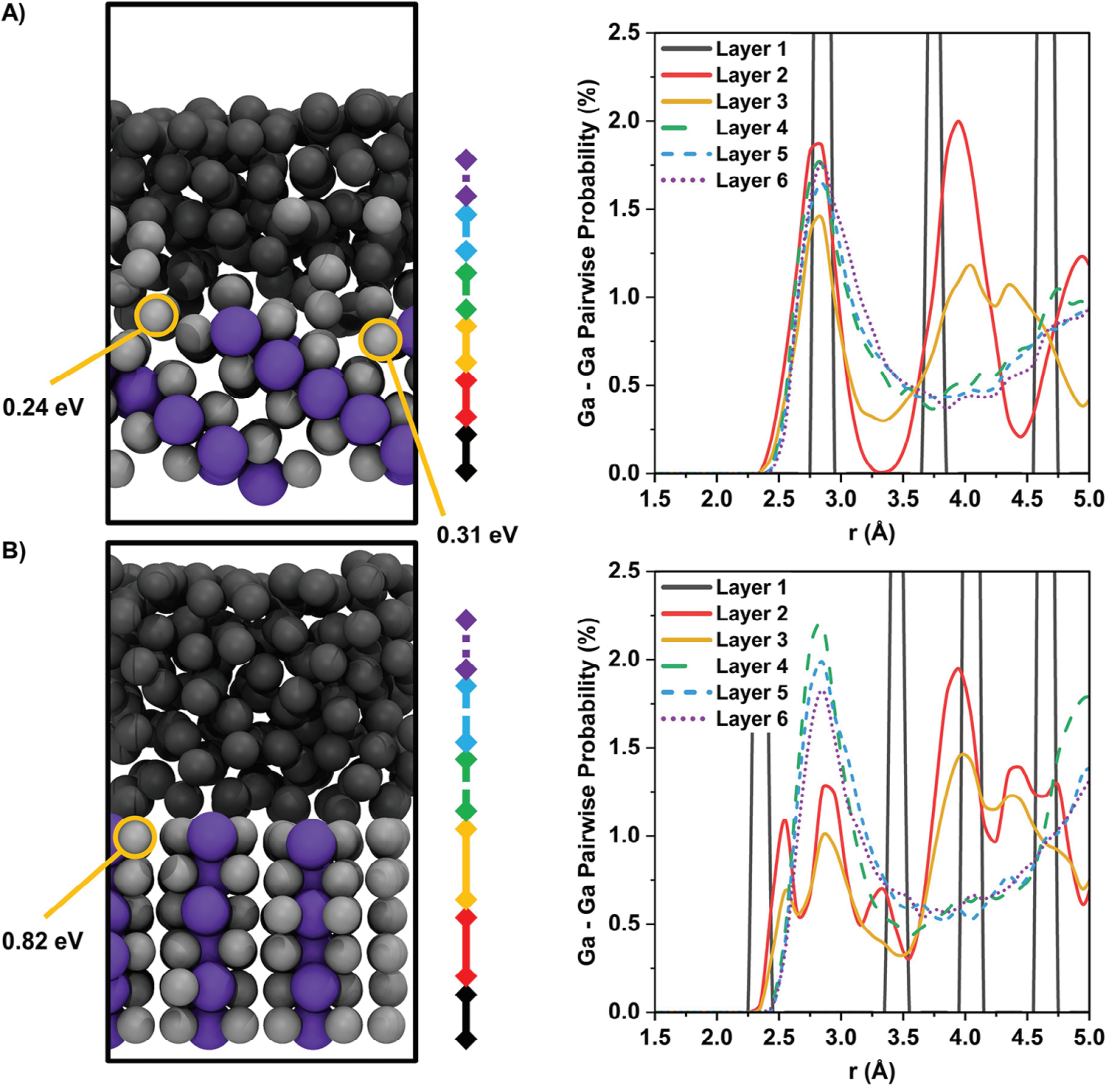
AIMD simulations of the [$0\bar{1}3$] and [$\bar{1}00$] facets of CuGa_2_ at 30 °C. Snapshots of the A) [$0\bar{1}3$] and B) [$\bar{1}00$] facets interfacing with liquid gallium after 200 ps (left). copper is colored purple and gallium from CuGa_2_ is colored light grey, while the liquid gallium is colored dark grey. Energies required to remove gallium atoms from their positions are highlighted in orange. Atomic pairwise probability distributions of gallium as a function of *z* position in the system are also shown (right). The *z* position within the system has been divided into defined layers based on atomic density profiles which are indicated by the colored bars to the right of the visual AIMD snapshot.

Ab initio molecular dynamics (AIMD) simulations were performed to provide a better understanding of the surface fluidization phenomenon in atomistic detail, as a function of CuGa_2_ facets. Based on the experimental findings, the [$0\bar{1}3$] and [$\bar{1}00$] facets were chosen as representatives for high‐ and low‐activity interfaces, respectively, for investigation and modeling. Each theoretical model comprised six layers of crystalline CuGa_2_ with the bottom layer frozen and the outermost layer consisting of 200 liquid gallium atoms. After only 200 ps of simulation at 30 °C, distinct differences emerged between the two systems (**Figure** **3**).

It can clearly be seen that at the interface of the [$0\bar{1}3$] facet, gallium atoms not in direct contact with copper spontaneously displace from the lattice and diffuse into the liquid bulk gallium during the AIMD simulations, creating vacancies. Although bulk gallium atoms were found in positions directly above the copper and in gaps left by the displaced surface gallium, these atoms retained their liquid characteristic, with frequent diffusion and exchanges (Figure 3A). In contrast, at the liquid‐solid interface of the [$\bar{1}00$] facet, no atoms were displaced from the lattice during the AIMD simulations (Figure 3B). These results can be rationalized based on the energy requirement to remove a single gallium atom from the surface. At the [$0\bar{1}3$] facet, the energy to remove a gallium atom not in direct contact with copper is 0.24, and 0.31 eV for an atom in direct contact with copper. However, at the [$\bar{1}00$] facet all gallium atoms are in direct contact with a copper atom, and the energy requirement to remove a single gallium atom is considerably higher at 0.82 eV. While no gallium atoms in direct contact with copper were observed to be displaced from the [$0\bar{1}3$] interface within the 200 ps simulation at 30 °C, the AIMD simulations showcase the early stages of interface liquefaction which over an experimental timeframe of many seconds, can result in the observed effect that reaches several atom layers of depth. Extending the timeframe of AIMD simulations to match those of the experimental observations is not feasible due to computational constraints. Instead, simulations of the same system at slightly higher temperatures (100 °C) were conducted in order to speed up the reactions involved in the interface liquefaction process. These higher temperature AIMD simulations revealed further melting initiated by the displacement of a gallium atom adjacent to copper. Although these simulations were performed at a higher temperature than what was used experimentally, they do provide an atomistic mechanism for CuGa_2_ surface liquefaction at the [$0\bar{1}3$] facet (Figure S8 and Video S10, Supporting Information). While the energy to displace a single gallium atom in direct contact with a copper atom is slightly higher, once a gallium atom in direct contact with copper is displaced, this is quickly followed by the dislodgement of the sub‐surface copper atom directly beneath it. This copper atom then rises to the interface and dislodges a single copper atom from the interfacial layer. For experimental timeframes of several seconds, it is quite conceivable that isolated as well as copper‐adjacent gallium atoms diffuse out of the solid lattice, resulting in vacancy formation that exceeds the stability threshold. This will result in lattice collapse and surface liquefaction. The [$\bar{1}00$] facet on the other hand was much more stable and no surface perturbation was observed within the duration of the simulations (Figure S9, Supporting Information). This finding aligns well with the observations reported in Figure 1E, where only minimal restructuring of the [$\bar{1}00$] facet was observed.

While the Cu‐Ga system investigated here is of most interest in the realm of catalysis, the tin‐based alloys are related to common solder formulations and as such understanding these systems and the surface chemistry that occurs during solidification is technologically significant in the electronic industry.^[^ ^41^, ^42^, ^43^ ^]^ Furthermore, many other alloys used for structural applications in aerospace, as well as common steels, feature well‐defined intermetallic compounds that precipitate from a liquid solvent metal during solidification.^[^ ^44^, ^45^, ^46^ ^]^ The considerable instability of certain intermetallic facets and the observed dynamic interface liquefaction at temperatures well below the formal melting point of the bulk intermetallic are expected to play a pivotal role in defining the ultimate microcrystalline structure and in turn the physical, chemical and electronic properties of the alloy.

## 3 Conclusion

Precipitation reactions of intermetallic compounds from alloy melts were imaged at high resolution enabling the direct observation of dynamic reversible equilibrium reactions. The surfaces of intermetallic compounds were discovered to alternate between a solid and liquid, effectively showcasing the forward (crystallization) and reverse (dissolution) reactions. This phenomenon affects the outer layers of atoms and can reach a depth of several nanometers, while the crystal interface liquefaction depth was found to be dependent on the exposed facet as well as the nature of the elements studied. This surface liquefaction occurred at remarkably low temperatures >200 K below the bulk melting point of the solid intermetallic. A degree of surface liquefaction was demonstrated for all samples that formed well‐defined intermetallic compounds, while alloy systems that did not form an intermetallic did not exhibit such effects.

Atomistic modeling was used to determine that for the model system Cu‐Ga, gallium atoms at the interface play a pivotal role in the melting process since they are weakly bound and can transition easily into the liquid phase, leaving behind vacancies that destabilize the lattice. The interface liquefaction effect presented here is also expected to occur in other alloy systems and may have technological implications if methods can be devised to modulate the interface liquefaction during the early stages of alloy solidification, where the metal is still largely liquid, but intermetallic compounds have begun to crystallize. Understanding and ultimately controlling the stability of these intermetallic interfaces, potentially by the addition of other solute metals, which are selected to act akin to a surfactant, may have significant industrial implications by enhancing metals used in applications in a variety of areas such as aerospace, additive manufacturing, and electronics.

## 4 Experimental Section

### Materials

Ga metal with a purity of 99.9% was procured from Indium Corporation, Sn metal with a purity of 99.99% from Testbourne Ltd, Bi metal with a purity of 99.9% from Roto Metals, Cu and Ag powder with a purity of 99.9% from Sigma–Aldrich, and anhydrous CH_3_COONa (NaOAc) with a purity of 99% from Sigma Aldrich. Additionally, ethanol with a purity of 99.5% from Thermo Fisher Scientific was also used.

### Preparation of Liquid Metal Alloys

To create the liquid metal alloys, specifically 5 wt.% CuSn, 10 wt.% AgSn, 5 wt.% CuGa, and 15 wt.% AgGa, the alloying process was conducted within a nitrogen glovebox to prevent oxidation. This involved blending the powders into the bulk metal at 400 °C using a mortar and pestle. The 10 wt.% BiGa alloy, on the other hand, was prepared by melting both Bi and Ga at 400 °C under ambient conditions in a beaker. Subsequently, all alloys were frozen at −80 °C and stored in a fridge at 5 °C in solid form.

### Nanodroplet Synthesis Method

The synthesis process began with breaking the solid metals into smaller quantities, each weighing ≈1.0 to 1.5 g.

For the high‐temperature sonication synthesis, NaOAc was employed as the solvent in a glass vial. ≈15.3 g of NaOAc was used, corresponding to roughly 10 mL in its molten state. The melting of the salt was carried out at 400 °C on a hotplate equipped with an aluminum heating block that had a void matching the shape of the glass vial, ensuring efficient heat transfer and uniform heating. Once all of the NaOAc was molten, ≈1 g metal was introduced and stirred into the mixture at 250 rpm using a magnetic stir bar. Following this, a SCIENTZ‐IID probe sonicator equipped with a 6 mm titanium tip was lowered into the molten NaOAc. After a brief thermalization period, sonication commenced for 30 min at 300 W power, following a 3‐second on, 3‐second off sonication pattern. Concurrently, magnetic stirring at 250 rpm continued.

Following sonication, the solution was allowed to cool and solidify. Once solidified and warm to the touch, the sample was dissolved in ≈500 mL of deionized water. The resulting particles were isolated through vacuum filtration using a 400 nm pore size PETE membrane with a 47 mm diameter from Sterlitech. After three washes with deionized water, the samples were redispersed in a 90 vol% ethanol‐water solution. This allowed for the extraction of a small amount of nanodroplets, which were then drop‐cast onto a Protochips e‐chip with a carbon membrane for heated TEM analysis.

### TEM Characterization

TEM videos were taken using JEOL F200, operating at an accelerating voltage of 200 kV. Gatan Digital Micrograph 3.43.3213.0 software suite was used for imaging and analysis with the use of Gatan Rio16 4k charge‐coupled device camera (model 1816). Furthermore, a heating/cooling setup is attached with a computer managing the rate at which temperature changes. For approaching temperatures, a heating rate of 0.48 °C/s was utilized, then the temperature remains stationary for the duration of ≈30 min to equilibrate. Allowing the usage of a screen capture setup to record various videos at these temperatures.

EDS was collected using an Oxford X‐Max^n^ 80T X‐ray spectrometer running the Aztec software suite. This allowed for elemental mapping of each droplet. Each statistical data was then plotted using Origin Pro 2023/2024.

### MD Simulations

The initial structure of intermetallic CuGa_2_ was taken from the Crystallography Open Database,^[^ ^47^, ^48^ ^]^ and six‐layer systems of the [$0\bar{1}3$] and [$\bar{1}00$] facets comprising 216 atoms were constructed using the Atomic Simulation Environment.^[^ ^49^ ^]^ The [$0\bar{1}3$] facet was constructed using a 6 × 2 supercell to give final dimensions of 16.980 Å × 20.608 Å, and the [$\bar{1}00$] facet a 4 × 3 supercell to give final dimensions of 16.00 Å × 17.51 Å. Initial classical MD simulations of 200 Ga atoms were carried out in rectangular boxes with the same *xy* dimensions as the facets using the MD code LAMMPS.^[^ ^50^ ^]^ Force field parameters for Ga were taken from the previous work.^[^ ^51^ ^]^ Following this initial equilibration, the bulk Ga was added to the facets, a 10 Å vacuum spacer was added to the systems, and 10 ps of AIMD simulation were run with all facet atoms frozen using the Vienna ab initio Simulation Package^[^ ^52^, ^53^ ^]^ with the projector‐augmented wave^[^ ^54^ ^]^ method, the PBE exchange‐correlation functional,^[^ ^55^ ^]^ an energy cutoff of 320.0 eV, a 4 fs time step, and the gamma point only for the k‐point grid. Following this relaxation of the bulk Ga over the facets, all CuGa_2_ layers, except the bottom one, were unfrozen, and an additional 200 ps of AIMD simulation were performed, in triplicate for each system, at 303.15 and 373.15 K to accelerate exploration of the configurational space. All analysis was carried out using VMD 1.9.3.^[^ ^56^ ^]^ Atoms were assigned to layers based on the minima in atomic density profiles calculated in the z dimension with a 1.5 Å bin width over the final 500 simulation snapshots (2 ps) for each system (Figure S10, Supporting Information).

### DSC Operation

Phase transition temperatures including melting (Tm) and solid‐solid transitions (Tss) were determined using a DSC TA Q200 calorimeter (TA Instruments). The equipment was calibrated using indium (TA instruments, Tm = 156 °C, ΔHf = 28.45 J g^−1^) and cyclohexane (Sigma–Aldrich, Tm = 8 °C) as standards. Measurements were performed in sapphire pans, under nitrogen atmosphere, in triplicate with sample sizes ≈10 mg. A heating rate of 10 °C min^−1^ was employed between the temperatures of −150 and 350 °C. The values and traces shown in this work are presented from the second run of the DSC cycle. Melting point (Tm) was considered as the onset of the peak.

## Conflict of Interest

The authors declare no conflict of interest.

## Supporting information

Supporting Information

Supplemental Video 1

Supplemental Video 2

Supplemental Video 3

Supplemental Video 4

Supplemental Video 5

Supplemental Video 6

Supplemental Video 7

Supplemental Video 8

Supplemental Video 9

Supplemental Video 10

## Data Availability

The data that support the findings of this study are available in the supplementary material of this article.
