# Supplementary material for: Spontaneous Liquefaction of Solid Metal–Liquid Metal Interfaces in Colloidal Binary Alloys
Source: Adv Sci (Weinh). 2024 May 5;11(26):2400147. doi: 10.1002/advs.202400147 (PMC11234468; doi:10.1002/advs.202400147)
Supplement: Supplementary file 1 — Supporting Information [file ADVS-11-2400147-s008.pdf]

ADVANCED SCIENCE
Open Access

Supporting Information

for *Adv. Sci.*, DOI 10.1002/advs.202400147

Spontaneous Liquefaction of Solid Metal–Liquid Metal Interfaces in Colloidal Binary Alloys

Caiden J. Parker, Karma Zuraiqi, Vaishnavi Krishnamurthi, Edwin LH Mayes, Pierre H. A. Vaillant, Syeda Saba Fatima, Karolina Matuszek, Jianbo Tang, Kourosh Kalantar-Zadeh, Nastaran Meftahi, Chris F. McConville, Aaron Elbourne, Salvy P. Russo, Andrew J. Christofferson*, Ken Chiang* and Torben Daeneke**

Spontaneous Liquefaction of Solid Metal–Liquid Metal Interfaces in Colloidal Binary Alloys

Caiden J. Parker[1], Karma Zuraiqi[1], Vaishnavi Krishnamurthi[1], Edwin LH Mayes[2], Pierre H. A. Vaillant[1], Syeda Saba Fatima[1], Karolina Matuszek[3], Jianbo Tang[4], Kourosh Kalantar-Zadeh[5], Nastaran Meftahi[6], Chris F. McConville[7], Aaron Elbourne[2], Salvy P. Russo[2,6], Andrew J. Christofferson[2,6]*, Ken Chiang[1]*, and Torben Daeneke[1]**

[1]School of Engineering, RMIT University, 3001 Melbourne, Australia

[2]School of Science, RMIT University, 3001 Melbourne, Australia

[3]School of Chemistry, Monash University, 3800 Clayton, Australia

[4]School of Engineering, University of New South Wales (UNSW), 2052 Sydney, Australia

[5]School of Chemical and Biomolecular Engineering, The University of Sydney, 2008 Sydney, Australia

[6]ARC Centre of Excellence in Exciton Science, School of Science, RMIT University, 3001 Melbourne, Australia

[7]Department of Physics, University of Warwick, Coventry CV4 7AL, United Kingdom

*Correspondence to torben.daeneke@rmit.edu.au, ken.chiang@rmit.edu.au, andrew.christofferson@rmit.edu.au, karma.zuraqi@rmit.edu.au

Keywords: liquid metal, liquefaction, nanodroplets, binary alloys, gallium colloid, $CuGa_2$

Supplementary Discussion 1: The melting point of Ga_2Cu

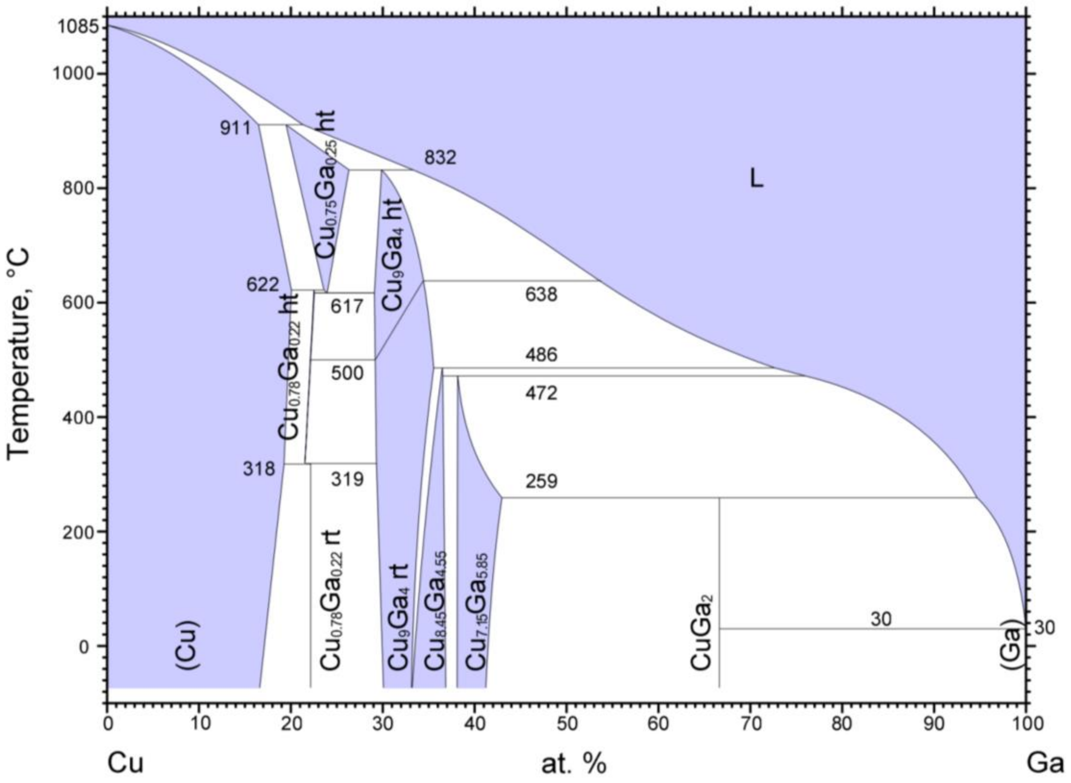

Figure S1: Cu-Ga phase diagram where the x-axis denotes atomic ratio of Ga to Cu in atomic percent and the y-axis denotes temperature of the system in degrees Celsius. The purple shaded region labeled 'L' (position upper right) indicates temperature and composition conditions where the alloy is liquid. Areas below the liquidus line indicate conditions where the sample is either completely or partially solid. Several defined intermetallic phases are indicated, with the $CuGa_2$ intermetallic being of particular significance for this work. The horizontal line labeled 259 indicates the maximum temperature for stable $CuGa_2$. For high Ga concentrations the liquidus line can be considered as the temperature dependent solubility of Cu in Ga. Reproduced with permission from Springer Materials.

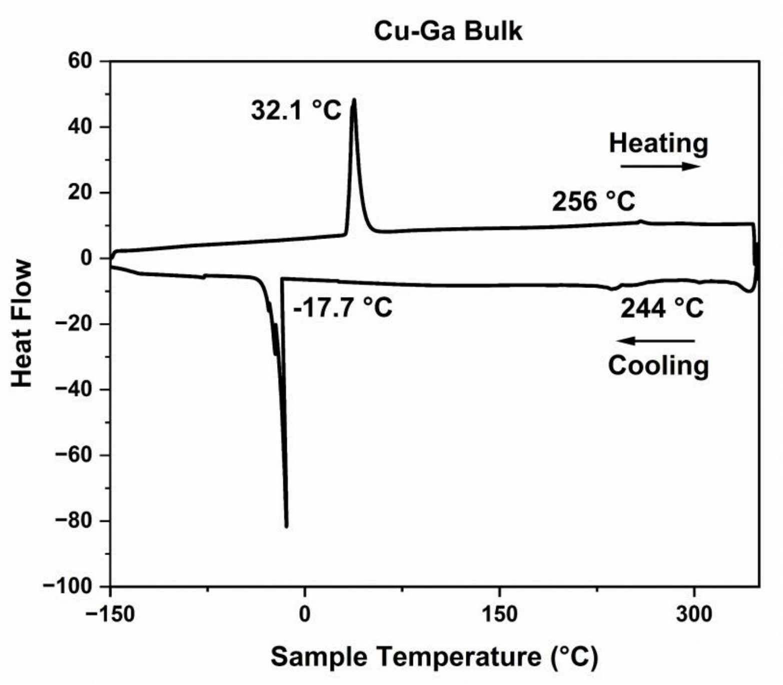

Figure S2: Differential scanning calorimetry of a Cu-Ga liquid metal alloy bulk sample, showcasing a slight increase in the melting point of Ga (onset at 32.1 °C in comparison to 29.8 °C for pure Ga), and a very slight peak with onset at 256 °C indicating the complete dissolution of the $CuGa_2$.

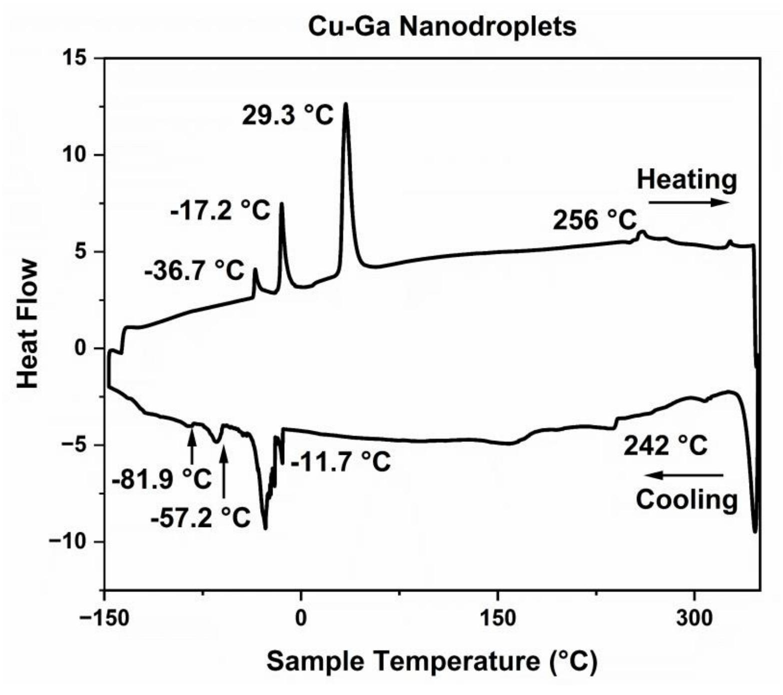

Figure S3: Differential scanning calorimetry of a Cu-Ga liquid metal alloy nanodroplet system showcasing three distinct Ga melting peaks indicating size effects. The dissolution of the intermetallic $CuGa_2$ is still observed with onset at 256 °C.

The phase transition behavior of the $CuGa_2$ intermetallic compound within the liquid Ga solvent was investigated using the differential scanning calorimetry (DSC). Both bulk and nanosized samples were investigated. During the heating scan for the bulk Cu-Ga sample (**Figure S2**) one distinct Ga melting peak is observed with onset at 32.1 °C while a subtle intermetallic melting peak is seen with onset at 256 °C. The slight increase of the Ga melting point from 29.8 to 32.1 °C reveals that the added Cu affects bonding structures within the bulk gallium sample resulting in a higher melting energy requirement. Upon cooling, the crystallization of both, the intermetallic compound and gallium occurred at temperatures lower than expected due to pronounced supercooling effects which are not uncommon in metallic systems.[1] Interestingly for nanocolloidal Cu-Ga (**Figure S3**), there were three distinct peaks with onsets at -36.7 °C, -17.2 °C, and 29.3 °C during the heating cycle. These peaks are likely caused by melting of various Ga phases. The peak at 29.3 °C can be associated with the melting of α-Ga, while the peak at -17.2 °C is most likely associated with the melting of β-Ga. The peak at -36.7 °C may be linked to γ-Ga.[2-3] Additionally, studies on gallium nanoparticles showed that several lower melting point phases can preferentially form for smaller sized particles. Since our nanodroplets are known to be a polydisperse system (size range 50 nm – 1 µm),[4] it is thus expected that populations of particles will crystallize in different phases that then melt at different temperatures. These melting points are further augmented by general size dependent melting point depression effects. At high temperatures, the melting of the $CuGa_2$ intermetallic can be observed at a peak onset of 256 °C, which corresponds to the peak observed in the bulk sample. While this melting transition is a minor feature in the DSC scan, it has been clearly identified and observed at that temperature during *in-situ* heating TEM experiments. The comparatively low intensity of the peak is the result of solid $CuGa_2$ only making up a small fraction of the overall sample at that temperature due to the higher solubility of Cu up to ~250 °C.

During the cooling cycle, the observed cluster of peaks below -11.7 °C is likely caused by the freezing of individual liquid metal nanodroplets. Crystallization is a stochastic process and may occur in different particles at different times, hence causing the observed feature.[5] The crystallization of the intermetallic commences at an peak onset of 242 °C which is comparable to the observations made for the bulk sample.

Overall, some effects of temperature depreciation due to operating in the nanometer regime are observed, while the low temperature regime is being dominated by effects caused due to gallium polymorphism in the nanometric regime.[6-9] The intermetallic $CuGa_2$ compound does not appear to be significantly impacted by size effects which may be in part due to the metallic nature of the surrounding medium. Melting point depreciation is usually caused by the increased impact of surface atoms, which lack the stabilizing effects of a complete network of neighbors, at the nanoscale. In a liquid metal environment, a delocalized metallic bond that spans across both the solvent and the intermetallic may counteract these effects. Since this work predominantly deals with the properties of intermetallics inside a liquid metal environment, bulk phase diagrams can still provide valuable guidance.

Supplementary Discussion 2: TEM beam heating effects

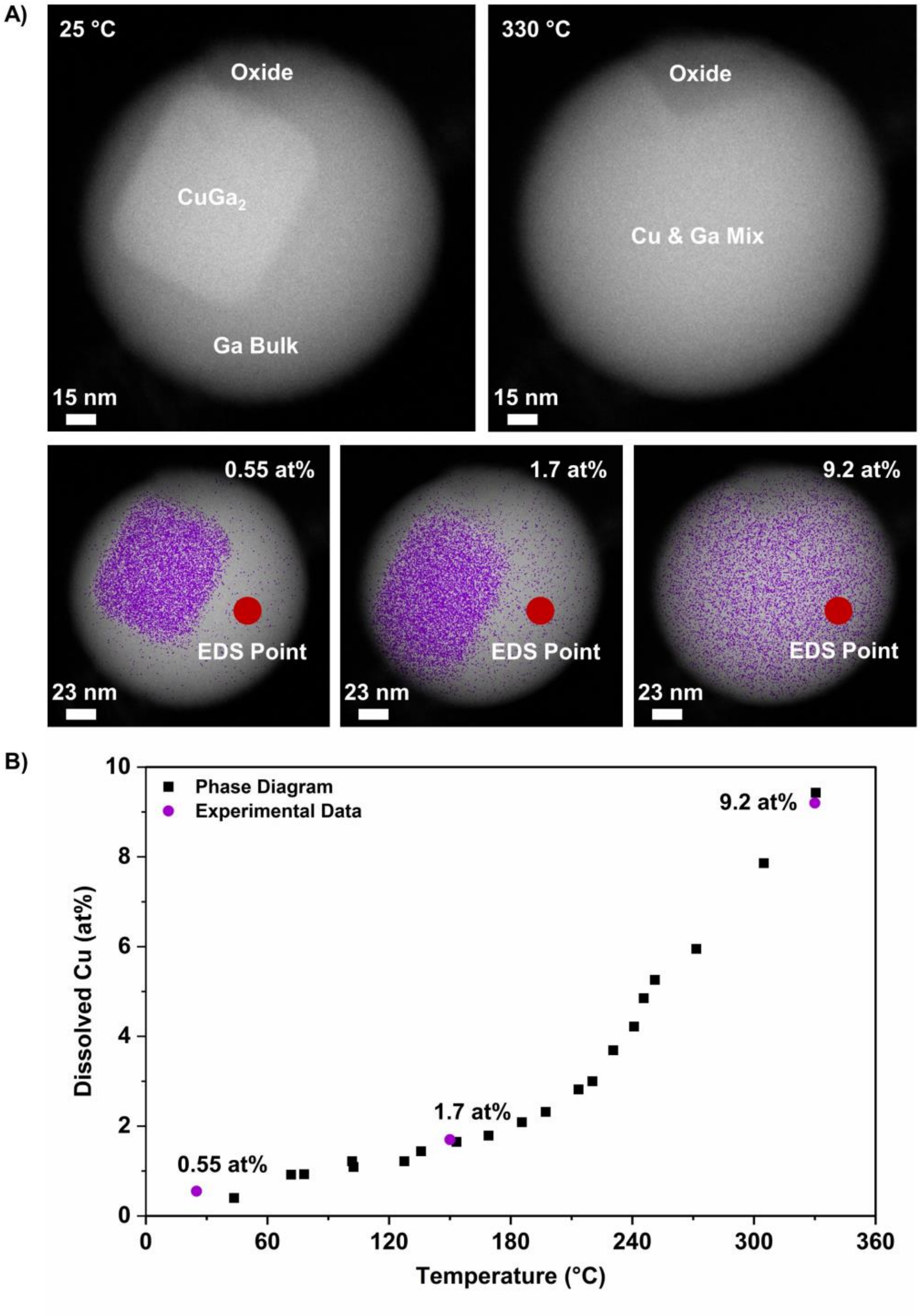

Figure S4: (A) Cu-Ga nanodroplet whereby the Cu intermetallic dissolves as the temperature increases until complete dissolution is achieved. **(B)** 3-point temperature calibration curve showing the measured Cu concentrations determined at the red indicated points in **Figure S3A** (purple data points accompanied by measured values), overlaid over the solid – liquid boundary manually extracted from the phase diagram shown in **Figure S1**, revealing that beam heating is negligible.

A 9 at% Cu-Ga metal in metal colloid sample was heated in the TEM until the intermetallic fully dissolved, thus creating a homogeneous liquid mixture of Cu and Ga atoms. During the heating process, the system traverses along the solid-liquid boundary of the phase diagram which is equivalent to the solubility limit of Cu in Ga. The concentration of Cu in the liquid portion of the droplet can then be measured using electron dispersive X-ray spectroscopy, providing a direct measure of the dissolution process.

Since the used *in-situ* system is well calibrated by the manufacturer, the dissolution process of the solid intermetallic can then be used as an internal standard to estimate beam heating. If beam heating was considerable, the measured dissolution curve would be shifted from the expected values based on reported solubility curves. **Figure S4A** shows several dark field images measured at different temperatures. The top row shows the room temperature sample as well as an image taken at high temperatures where the intermetallic is fully dissolved. During heating, the solid fraction slowly reduces in size until it is fully dissolved. The temperature at which the entire intermetallic has become molten is in agreement with the DSC measurements discussed above and the phase diagram shown in **Figure S1**. **Figure S4B** shows three individually measured concentrations at specified temperatures (purple) superimposed onto the solubility limit of the Cu-Ga system (black), indicating that beam heating is negligible.

A more accurate estimation of beam heating can be achieved using published equations, such as the one listed below.[10-11]

$$I\Delta E\left(\frac{t}{\lambda}\right) = 4\pi kt\frac{T - T_0}{0.58 + 2\ln\left(\frac{2R_0}{d}\right)} \qquad (1)$$

Here I is the incident beam current in C/s, ΔE is the average energy loss in J/C, t is the sample thickness in nm, λ is the electron mean free path in nm, k is the thermal conductivity of the

sample in W/mK, d is the incident electron beam diameter in nm, R_0 is the travelling distance of heating in nm, and T is the sample temperature in K.

Upon rearranging equation (1), we can get T as the subject which is the temperature of the sample after thermal equilibrium is achieved.

$$\Delta T = \frac{I\Delta E\left(\frac{t}{\lambda}\right)\left(0.58 + 2\ln\left(\frac{2R_0}{d}\right)\right)}{4\pi kt} \qquad (2)$$

After utilizing equation (2) and applying either measured or literature values for the relevant variables, we arrive at an estimated temperature increase of 0.04 °C which confirms that beam heating is indeed negligible.

Aside from measuring the dissolution process, TEM based elemental mapping also indicates where the surface oxide layers reside. All synthesized nanodroplets contain an oxide layer that spontaneously forms in oxygen containing environments (i.e. Air). Gallium as well as tin based metals tend to oxidize *via* the Cabrera-Mott process, forming a 1-2 nm thick oxide layer.[12-13] Under almost all circumstances the intermetallic compound was found to be preferentially positioned inside the droplet closely situated to the surface oxide. This could be caused by preferential nucleation at the interface or due to weak surface interactions (i.e. van der Waals forces). Throughout this work, the intermetallic compound has been carefully studied in areas where it is detached from the oxide in order to minimize any local impact of the interface.

Supplementary Discussion 3: Laplace pressure

The Laplace pressure describes the relationship of the surface tension of a liquid material to the internal pressure of the system. For nanodroplets this pressure can be considerable and it is critical to consider the Laplace pressure inside such systems.[14] The equation describing the Laplace pressure inside droplets is:

$$\Delta \mathrm{P} = \gamma_{L-V}\left(\frac{2}{\mathrm{R}}\right) \qquad (3)$$

Where P is the internal pressure in N/m^2, $\gamma_{L\text{-}V}$ is surface tension of the material in N/m, and R is the radius of the nanodroplet in m.

Utilizing equation (3) for the two droplets studied in **Figure 1E** of the main text with the sizes of 109 nm and 179 nm, and assuming a surface tension of 0.708 N/m,[15] leads to pressures of 260 bar and 158 bar respectively. As can be seen, the difference of the internal pressure is considerable and can explain the variation in surface reactivity to a degree. Please note, the absolute values of the internal pressure are highly dependent on the used value for the surface tension which in turn depends on the interfacial chemistry of the nanodroplets (*i.e.* the quality of the oxide layer).

Supplementary Discussion 4: Compositional Determination

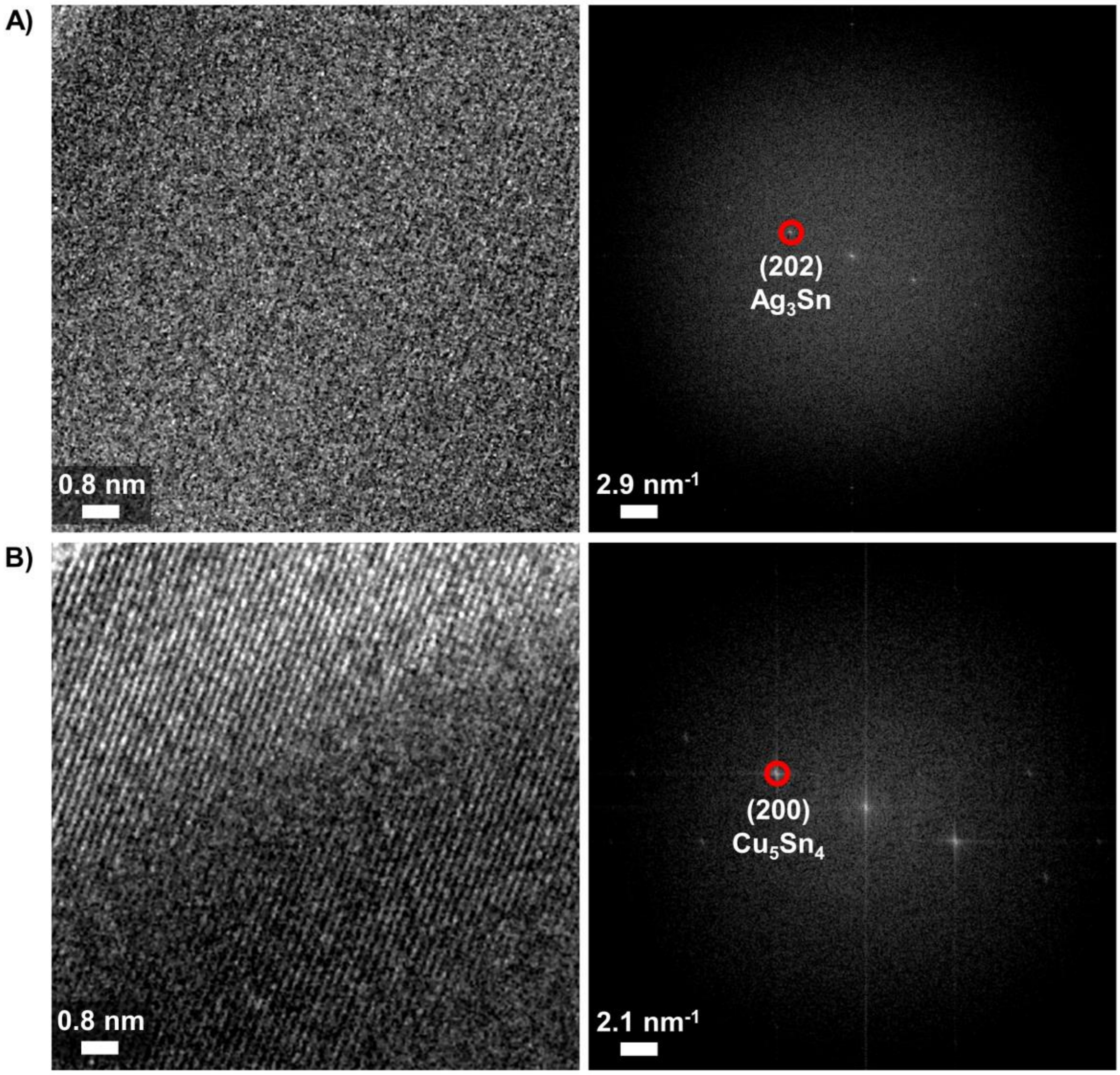

Figure S5: Crystal lattice of **(A)** Ag_3Sn[16] and **(B)** Cu_5Sn_4[17] with an FFT showcasing the crystal structure which confirmed their planar orientation and thus the intermetallic compound.

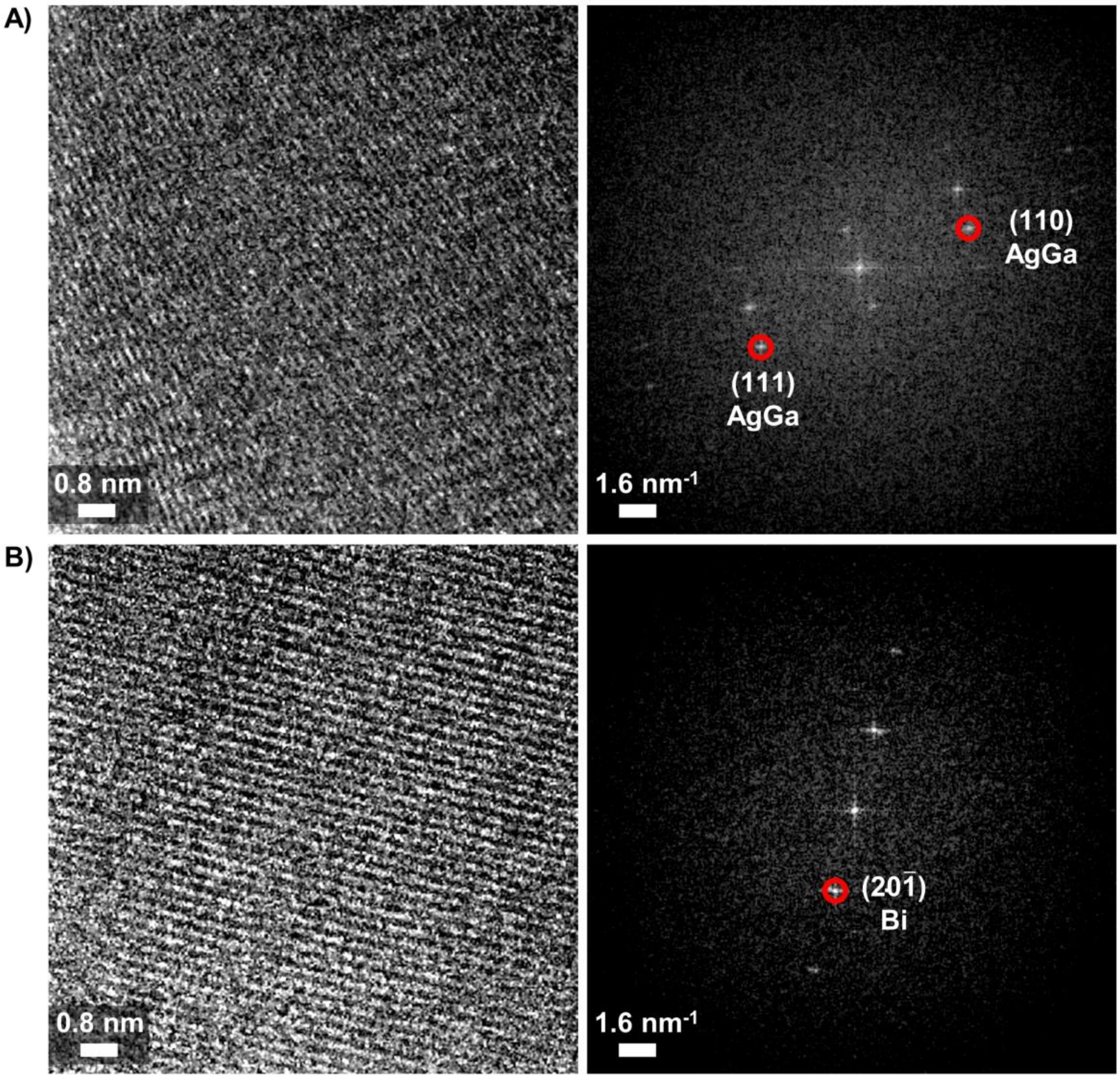

Figure S6: Crystal lattice of **(A)** AgGa[18] and **(B)** Bi[19] with an FFT showcasing the crystal structure which confirmed their planar orientation and thus the intermetallic or non-intermetallic compound.

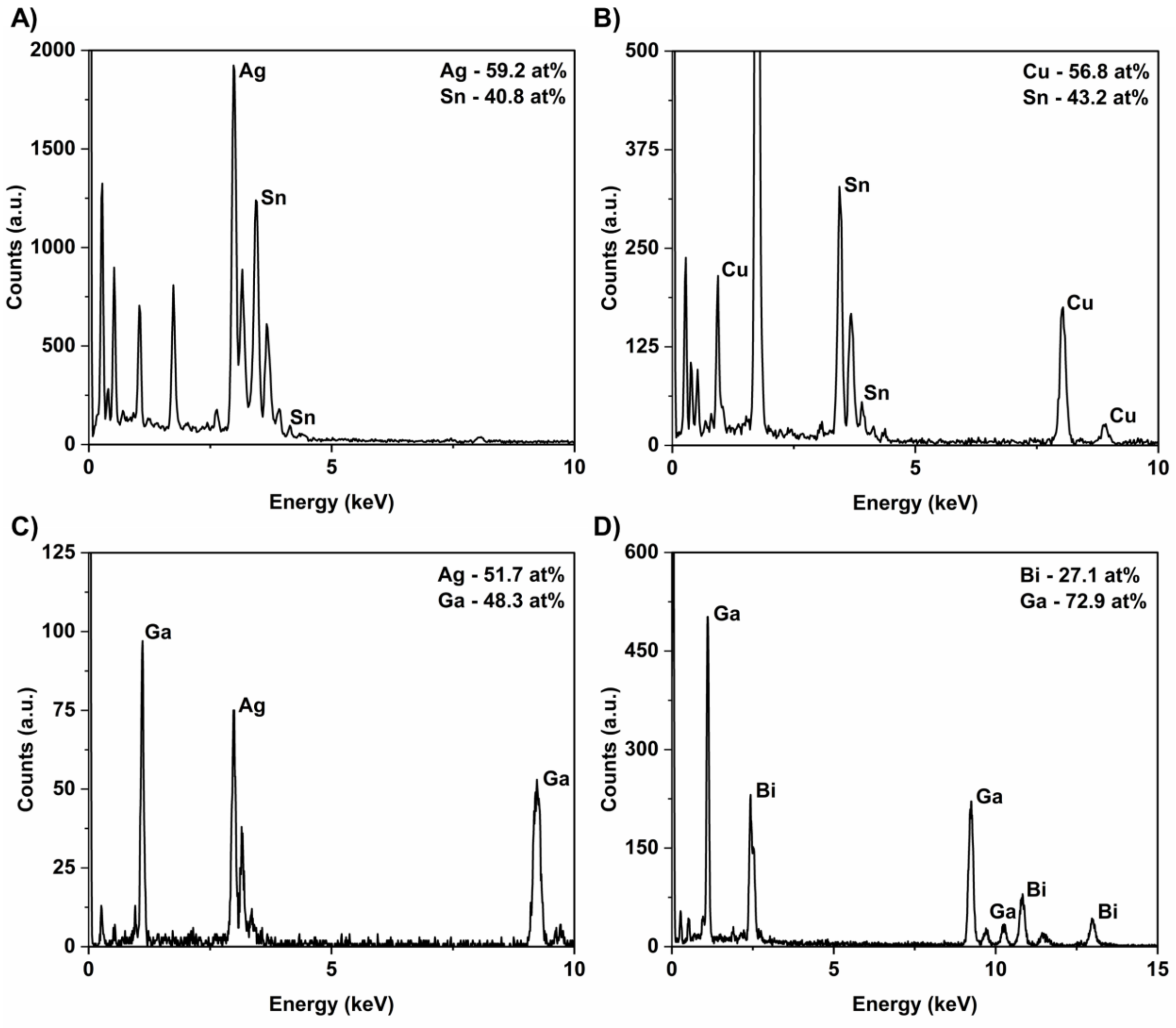

Figure S7: EDS spectrum data showcasing the atomic percentage and hence the compositional analysis of **(A)** Ag_3Sn, **(B)** Cu_5Sn_4, **(C)** AgGa, and **(D)** Bi, whereby the percentages are similar to their stoichiometric ratios thus confirming intermetallic composition.

The determination of the composition for the intermetallic is carried out by analyzing the Fast Fourier-Transform (FFT) of atomic resolution TEM images that show the lattice fringes. Here the lattice planes of the crystalline solid have been determined and cross referenced to relevant crystal databases (**Figure S4 & S5**), facilitating the identification of the most likely intermetallic phase. The process was facilitated by consulting published phase diagrams which can assist in identifying the most likely compositions. In addition to crystallographic analysis, the samples were further analyzed via energy dispersive X-ray spectroscopy (EDS). Within **Figure S6**, each of the intermetallic compositions were measured using the elemental maps

shown in Figure 2 of the main text. The elemental compositions were measured on the solid portion (i.e. the crystalline core) and towards the edge of the droplet in order to reduce the likelihood of significant portions of liquid metal being above or below the solid crystal. This compositional data is overall congruent with the determined intermetallic stoichiometry of the individual intermetallics. However, it is worth noting that inaccuracies may arise due to fluorescent scattering effects where nearby sample may skew the results, and spectral overlap between the elements.

AIMD Supplementary Images

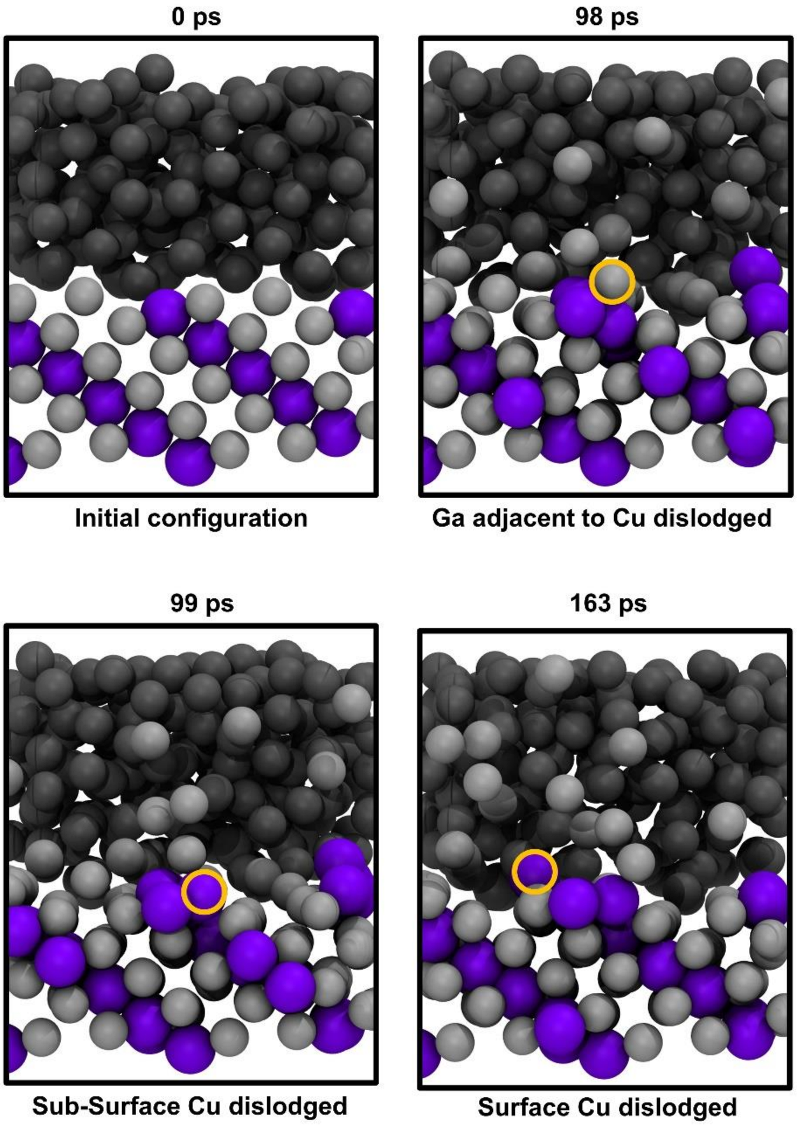

Figure S8: Mechanism for [0$\bar{1}$3] facet melting from AIMD simulations run at 100 °C. Specific atom movements are highlighted in orange.

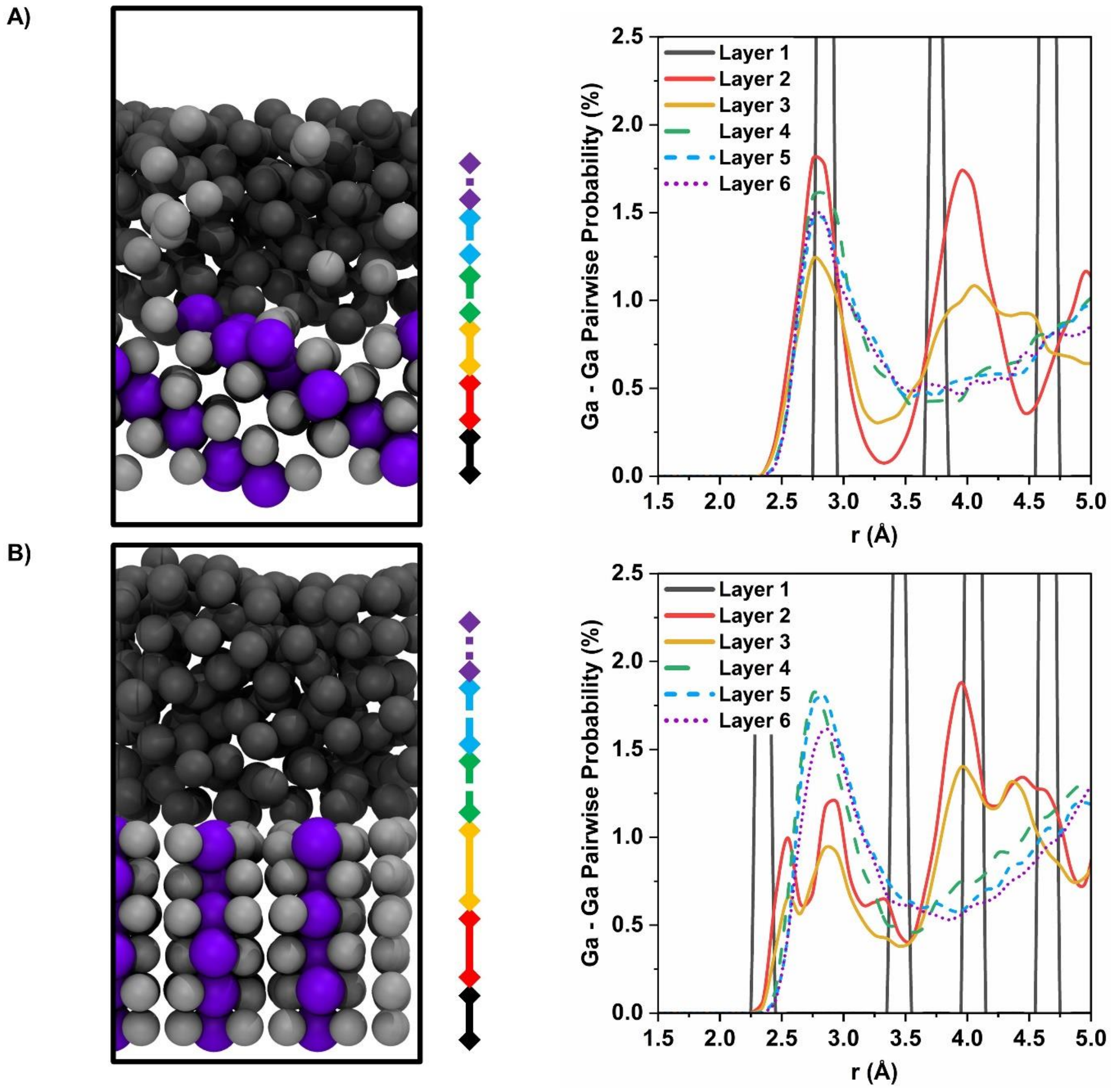

Figure S9: AIMD simulations of the **(A)** [0$\bar{1}$3] and **(B)** [$\bar{1}$00] facets of Ga_2Cu at 100 °C. Snapshots of the **(A)** [0$\bar{1}$3] and **(B)** [$\bar{1}$00] facets interfacing with liquid gallium after 200 ps (left). Copper is colored purple and gallium from Ga_2Cu is colored light grey, while the liquid gallium is colored dark grey. Energies required to remove gallium atoms from their positions are highlighted in orange. Atomic pairwise probability distributions of gallium as a function of *z* position in the system are also shown (right). The *z* position within the system has been divided into defined layers which are indicated by the colored bars to the right of the visual AIMD snapshot.

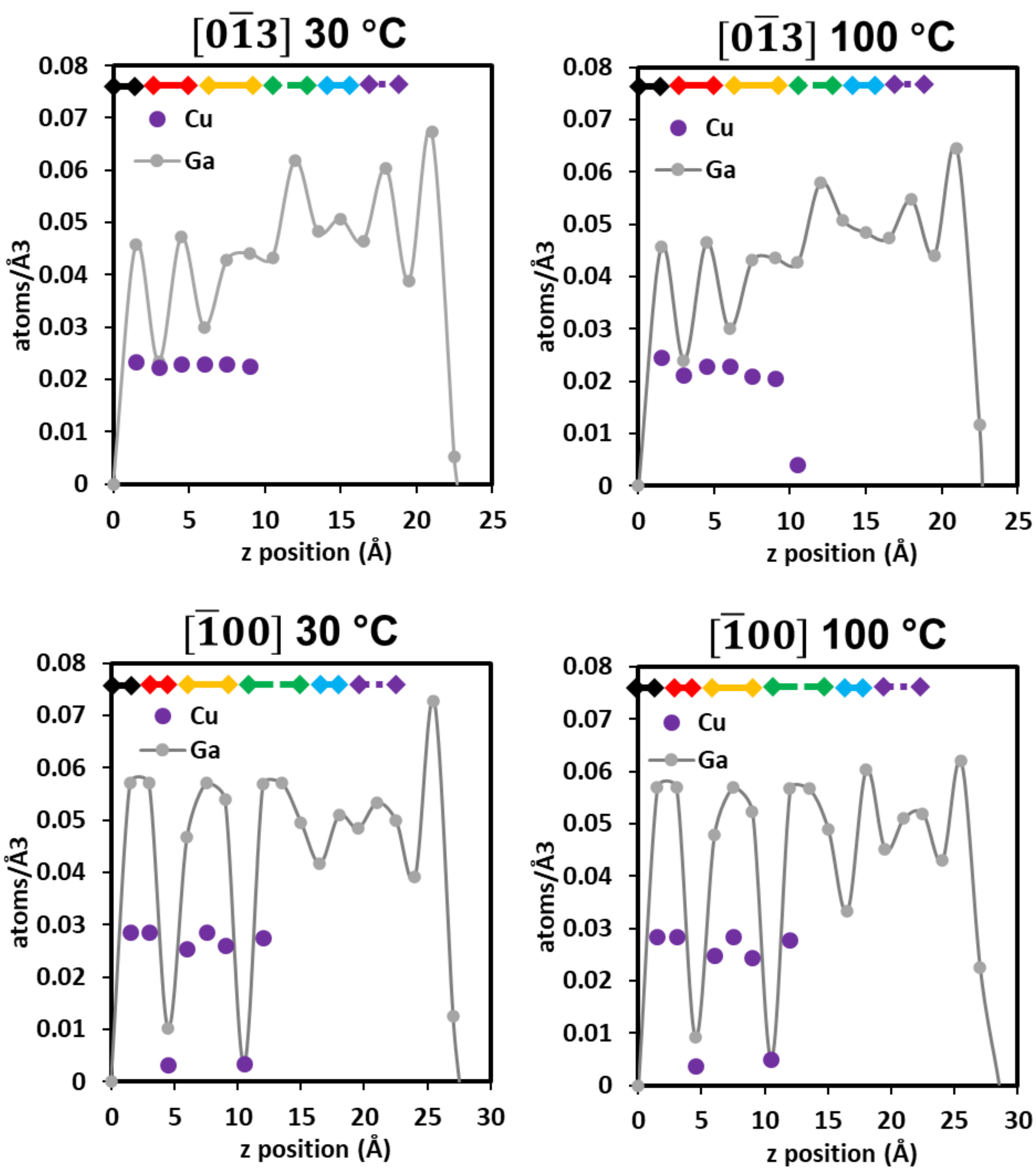

Figure S10: Atomic density profiles for Ga and Cu atoms from the AIMD simulations, calculated over the final 2 ps of each simulation. In each case the first layer is defined as the bottom layer that is frozen in AIMD simulation.

Unaltered Figures

Each Figure that is listed here has been altered in the main manuscript to provide visual aid. This enhances the readers' experience if they lack access to the supplementary videos and makes it easier to follow the discussion.

Note, it is highly recommended to watch the supplementary videos since they provide a clearer picture as to what is happening.

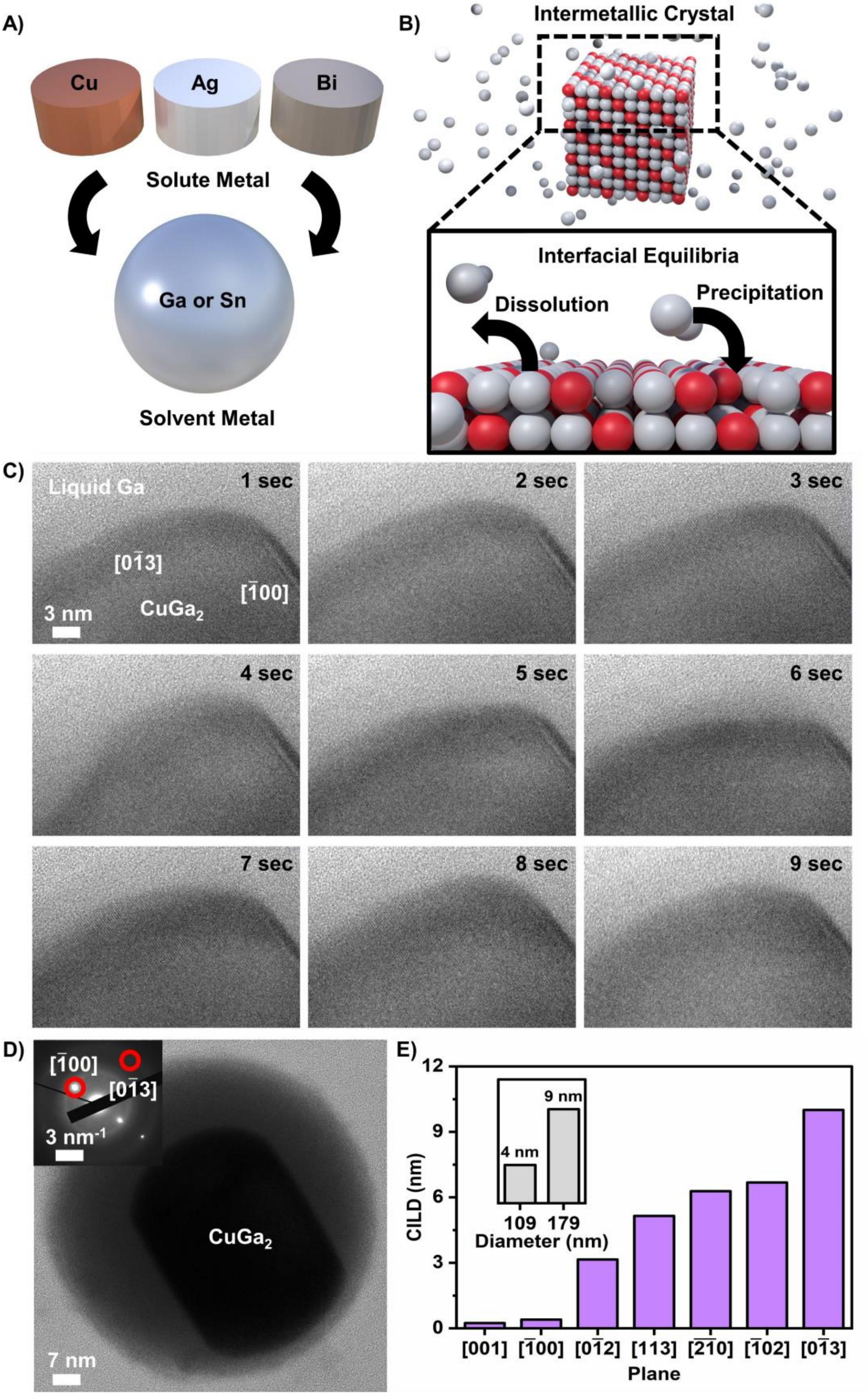

Figure S11: (A) Material selection process showing the solute metals (top) and the liquid metal solvents (bottom), **(B)** Schematic of an intermetallic colloidal particle undergoing an

equilibrium reaction whereby liquid metal atoms migrate into solution (dissolution) to then later be replaced by another liquid metal atom from the bulk solution (precipitation). **(C)** Time series for dynamic movement of the Ga_2Cu crystal interface in a liquid metal environment. **(D)** Bright-field TEM image showcasing the Cu-Ga metal in metal colloid structure, with selected area electron diffraction (SAED) shown as an inset, and **(E)** Graphical representation demonstrating facet dependence and size dependence (inset) of the peak equilibrium displacement (PED).

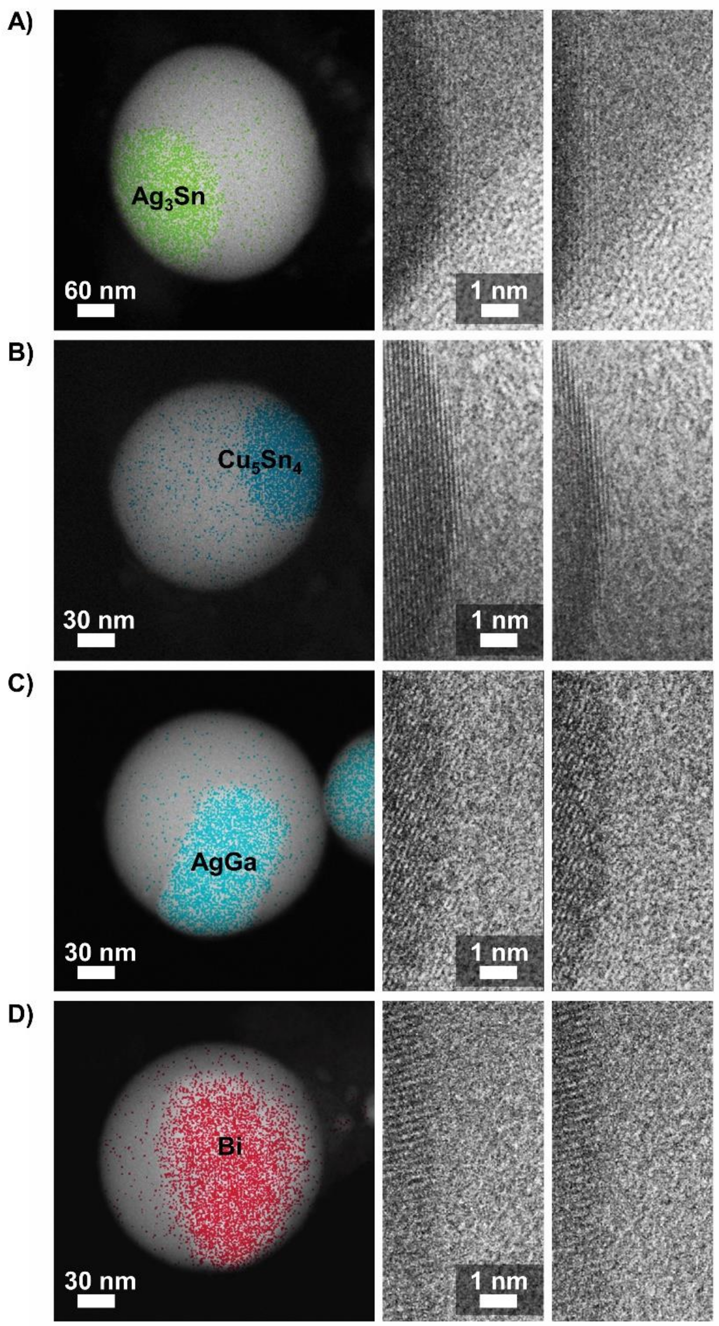

Figure S12: Dynamic movement investigated in **(A)** Ag-Sn, **(B)** Cu-Sn, **(C)** Ag-Ga, and **(D)** Bi-Ga. The left column shows dark field images of the studied droplets. The colored elemental map highlights the distribution of the respective added solute metal measured *via* electron dispersive X-ray spectroscopy. The composition of the solid colloidal particle has been determined *via* electron diffraction studies. The middle and right-hand columns show high-resolution images of an outer facet exposed to the liquid metal liquor.

Videos

Video V1: Cu-Ga dynamic movement directly attached to Figure 1c of main manuscript.

Video V2: Cu-Ga dissolution video from room temperature to 280°C

Video V3: Cu-Ga dynamic movement zoomed out.

Video V4: Cu-Ga dynamic movement on various facets.

Video V5: Cu-Ga dynamic movement with two different nanodroplet sizes with the same facet.

Video V6: Cu-Sn dynamic movement directly attached to Figure 2a of main manuscript.

Video V7: Ag-Sn dynamic movement directly attached to Figure 2b of main manuscript.

Video V8: Ag-Ga dynamic movement directly attached to Figure 2c of main manuscript.

Video V9: Bi-Ga dynamic movement directly attached to Figure 2d of main manuscript.

Video V10: AIMD modeling of the $[0\bar{1}3]$ surface at 100 ºC

References

[1] J. Ma, P. Zhang, *Materials Advances* **2021**, *2* (22), 7437, https://doi.org/10.1039/d1ma00601k.
[2] H. He, G. T. Fei, P. Cui, K. Zheng, L. M. Liang, Y. Li, L. De Zhang, *Physical Review B* **2005**, *72* (7), 073310, https://doi.org/10.1103/PhysRevB.72.073310.
[3] J. Mingear, Z. Farrell, D. Hartl, C. Tabor, *Nanoscale* **2021**, *13* (2), 730, https://doi.org/10.1039/D0NR06526A.
[4] C. J. Parker, V. Krishnamurthi, K. Zuraiqi, C. K. Nguyen, M. Irfan, F. Jabbar, D. Yang, M. P. Aukarasereenont, E. L. H. Mayes, B. J. Murdoch, A. Elbourne, K. Chiang, T. Daeneke, *Adv. Funct. Mater.* **2023**, 2304248, https://doi.org/10.1002/adfm.202304248.
[5] J. Liu, L. Song, Z. He, S. Wang, W. Zhang, H. Yang, F. Li, S. Li, J. Wang, H. Xiao, D. Xu, Y. Liu, Y. Wu, J. Q. Wang, X. Shui, Y. C. Hu, J. Shang, R. W. Li, *Small* **2023**, https://doi.org/10.1002/smll.202305798.
[6] F. Gao, Z. Gu, in Springer International Publishing, **2016**.
[7] M. Yarema, M. Wörle, M. D. Rossell, R. Erni, R. Caputo, L. Protesescu, K. V. Kravchyk, D. N. Dirin, K. Lienau, F. Von Rohr, A. Schilling, M. Nachtegaal, M. V. Kovalenko, *Journal of the American Chemical Society* **2014**, *136* (35), 12422, https://doi.org/10.1021/ja506712d.
[8] P. Antoniammal, D. Arivuoli, *Journal of Nanomaterials* **2012**, *2012*, 415797, https://doi.org/10.1155/2012/415797.
[9] V. B. Kumar, Z. E. Porat, A. Gedanken, *Journal of Thermal Analysis and Calorimetry* **2015**, *119* (3), 1587, https://doi.org/10.1007/s10973-015-4402-x.
[10] L. Reimer, in Springer Berlin Heidelberg, **1998**.
[11] Y. Liao, Heat Generation/Temperature Increase by Electron Irradiation. Practical Electron Microscopy and Database: **2007**.
[12] A. Goff, P. Aukarasereenont, C. K. Nguyen, R. Grant, N. Syed, A. Zavabeti, A. Elbourne, T. Daeneke, *Dalton Trans.* **2021**, *50* (22), 7513, https://doi.org/10.1039/d0dt04364h.
[13] P. Aukarasereenont, A. Goff, C. K. Nguyen, C. F. McConville, A. Elbourne, A. Zavabeti, T. Daeneke, *Chemical Society Reviews* **2022**, *51* (4), 1253, https://doi.org/10.1039/D1CS01166A.
[14] M. Losurdo, A. Suvorova, S. Rubanov, K. Hingerl, A. S. Brown, *Nat. Mater.* **2016**, *15* (9), 995, https://doi.org/10.1038/nmat4705.
[15] S. C. Hardy, *J. Cryst. Growth* **1985**, *71* (3), 602, https://doi.org/https://doi.org/10.1016/0022-0248(85)90367-7.
[16] C. W. Fairhurst, J. B. Cohen, *Acta Crystallographica Section B* **1972**, *28* (2), 371, https://doi.org/doi:10.1107/S0567740872002432.
[17] P. Villars, K. Cenzual, Cu3Sn Crystal Structure: Datasheet from "PAULING FILE Multinaries Edition – 2022" in SpringerMaterials (https://materials.springer.com/isp/crystallographic/docs/sd_0451229), https://materials.springer.com/isp/crystallographic/docs/sd_0451229, accessed: 3 Sept, **2023**.
[18] P. Feschotte, P. Bass, *Journal of the Less Common Metals* **1991**, *171* (1), 157, https://doi.org/https://doi.org/10.1016/0022-5088(91)90272-6.
[19] R. M. Brugger, R. B. Bennion, T. G. Worlton, *Physics Letters A* **1967**, *24* (13), 714, https://doi.org/https://doi.org/10.1016/0375-9601(67)90230-7.
